# Nursing Resources as Mediators of COVID-19 Mortality in High Medicaid-Serving Hospitals

**DOI:** 10.1093/geroni/igaf122.3820

**Published:** 2025-12-31

**Authors:** Sherif Olanrewaju, Ann Kutney-Lee, Daniela Gollinelli, Margo Brooks Carthon

**Affiliations:** University of Pennsylvania, Philadelphia, Pennsylvania, United States; University of Pennsylvania, Philadelphia, Pennsylvania, United States; University of Pennsylvania, Philadelphia, Pennsylvania, United States; University of Pennsylvania, Philadelphia, Pennsylvania, United States

## Abstract

The COVID-19 pandemic exposed profound disparities within the United States’ healthcare system, notably highlighting significant variations in mortality outcomes across different patient groups, particularly among older adults and those from economically disadvantaged populations. Higher rates of mortality among patients hospitalized with COVID-19 were noted in hospitals that cared for larger proportions of Medicaid-insured patients. While some have attributed these differences to hospital and patient characteristics, a potential factor that has been largely underexplored is whether nursing resources, including staffing levels and the quality of the work environment, were associated with the observed disparities in outcomes. Using an innovative hospital benchmarking methodology, this cross-sectional study examined whether nurse staffing and the work environment acted as mediators of the relationship between high Medicaid-serving hospitals and COVID-19 mortality. Using linked nurse survey, administrative hospital, and patient discharge data from 2020, our study included 109,900 patients (mean age=63) hospitalized with COVID-19 in 118 hospitals located across New York. Findings from the hospital-level analysis suggested that nurse staffing and the nurse work environment act as partial mediators of the relationship between high Medicaid-serving status and COVID-19 mortality, with nurse staffing demonstrating the largest mediation effect (28%). Our findings suggest that bolstering nursing resources in hospitals that serve higher proportions of Medicaid-patients may be an important key to reducing disparities and planning for future public health emergencies. Insights gained from this research could significantly inform healthcare policy decisions and resource allocation strategies, particularly in hospitals serving high proportions of older adults insured by Medicaid.

